# Development of a Dementia Case Management Information System App: Mixed Methods Study

**DOI:** 10.2196/56549

**Published:** 2024-09-23

**Authors:** Huei-Ling Huang, Yi-Ping Chao, Chun-Yu Kuo, Ya-Li Sung, Yea-Ing L Shyu, Wen-Chuin Hsu

**Affiliations:** 1 Department of Gerontology and Health Care Management College of Nursing Chang Gung University of Science and Technology Taoyuan City Taiwan; 2 Dementia Center Department of Neurology Taoyuan Chang Gung Memorial Hospital Taoyuan Taiwan; 3 Geriatric and Long-Term Care Research Center Chang Gung University of Science and Technology Taoyuan Taiwan; 4 Department of Computer Science and Information Engineering Chang Gung University Taoyuan Taiwan; 5 Department of Otolaryngology-Head and Neck Surgery Chang Gung Memorial Hospital at Linkou Taoyuan Taiwan; 6 School of Nursing College of Medicine Chang Gung University Taoyuan Taiwan; 7 Healthy Aging Research Center Chang Gung University Taoyuan Taiwan

**Keywords:** case management, dementia, health information systems, mobile apps, user needs, mobile phone

## Abstract

**Background:**

Case managers for persons with dementia not only coordinate patient care but also provide family caregivers with educational material and available support services. Taiwan uses a government-based information system for monitoring the provision of health care services. Unfortunately, scheduling patient care and providing information to family caregivers continues to be paper-based, which results in a duplication of patient assessments, complicates scheduling of follow-ups, and hinders communication with caregivers, which limits the ability of case managers to provide cohesive, quality care.

**Objective:**

This multiphase study aimed to develop an electronic information system for dementia care case managers based on their perceived case management needs and what they would like included in an electronic health care app.

**Methods:**

Case managers were recruited to participate (N=63) by purposive sampling from 28 facilities representing two types of community-based dementia care centers in Taiwan. A dementia case management information system (DCMIS) app was developed in four phases. Phase 1 assessed what should be included in the app by analyzing qualitative face-to-face or internet-based interviews with 33 case managers. Phase 2 formulated a framework for the app to support case managers based on key categories identified in phase 1. During phase 3, a multidisciplinary team of information technology engineers and dementia care experts developed the DCMIS app: hardware and software components were selected, including platforms for messaging, data management, and security. The app was designed to eventually interface with a family caregiver app. Phase 4 involved pilot-testing the DCMIS app with a second group of managers (n=30); feedback was provided via face-to-face interviews about their user experience.

**Results:**

Findings from interviews in phase 1 indicated the DCMIS framework should include unified databases for patient reminder follow-up scheduling, support services, a health education module, and shared recordkeeping to facilitate teamwork, networking, and communication. The DCMIS app was built on the LINE (LY Corporation) messaging platform, which is the mobile app most widely used in Taiwan. An open-source database management system allows secure entry and storage of user information and patient data. Case managers had easy access to educational materials on dementia and caregiving for persons living with dementia that could be provided to caregivers. Interviews with case managers following pilot testing indicated that the DCMIS app facilitated the completion of tasks and management responsibilities. Some case managers thought it would be helpful to have a DCMIS desktop computer system rather than a mobile app.

**Conclusions:**

Based on pilot testing, the DCMIS app could reduce the growing challenges of high caseloads faced by case managers of persons with dementia, which could improve continuity of care. These findings will serve as a reference when the system is fully developed and integrated with the electronic health care system in Taiwan.

## Introduction

The global population of persons living with dementia currently exceeds 55 million. The progressive decline in cognitive impairment and symptoms associated with dementia has a significant impact on the quality of life for patients and families [1] and a global economic cost of US $1.3 trillion [2]. Nearly 50% of the health care costs for persons with dementia stem from informal caregivers such as family members or close friends [2]. Case managers for persons living with dementia who are cared for by informal caregivers provide comprehensive information on medical treatment, caregiving techniques, and social resources, and face additional challenges when providing services for persons with early onset dementia [3]. Case management for these patients involves coordinating assessments, follow-up evaluations, and care, which requires providing caregivers with information about navigating support services available to the patient, as well as the caregiver [3-5].

Taiwan initiated “Long-Term Care 2.0,” which prioritizes dementia care, and within this framework, the focus is on enhancing case management by providing consultations, advice, and support on a regular basis. By 2025, the targeted goal for the percentage of persons living with dementia receiving care facilitated by case managers will exceed 80% [6]. Case management encompasses the coordination and linking of care service resources, which requires case managers to provide regular comprehensive and continuous care services, including monthly consultations, referrals, and service resources, which is based on the severity of dementia, their caregiving needs, and input from caregivers [7]. Currently, each dementia care case manager oversees a caseload with a minimum of 150 patients, which can sometimes exceed 300.

Case managers at community-based dementia care centers use the Dementia Care Service Management System, offered by the Ministry of Health and Welfare, for registration of cases and medical expense reimbursement [7]. However, there is no electronic health care system for managing follow-ups, and integration of health care with health education information is lacking. This leads to duplicate assessments, discontinuities in health education interventions, and the inability to provide timely services. Case managers with no experience or knowledge about certain aspects of dementia care are impacted by the lack of an accessible health education database, requiring them to spend time conducting internet-based searches or reading paper-based health education brochures, which affects their efficiency and confidence [8]. The absence of an established robust information support system that can facilitate data management and easy access to information and education material on dementia care leads to inefficient service and impedes the ability to provide continuous and high-quality case management.

IT in the form of mobile apps, internet-based interventions, and eHealth has been demonstrated to provide support for caregivers of persons with dementia. Mobile apps have been demonstrated to benefit family caregivers of persons living with dementia to help manage their relative’s disruptive behaviors [9] and reduce caregiver burden [10], and an internet-based supportive intervention can reduce caregivers’ depressive symptoms and stress and increase self-efficacy [11]. A systematic review found that eHealth benefited health care staff in long-term care facilities by providing remote consultations for comprehensive assessments and decision-making [12]. Therefore, it should be feasible to facilitate dementia case management with an IT-based support app.

A decision support mobile app for dementia care case managers was designed to increase confidence in their management role. Although overall confidence did not improve compared with a control group, case managers highly recommended the use of the app [8]. This finding and those of studies on caregivers of persons with dementia suggest electronic health care could have significant benefits for dementia care case managers. However, there is no available technology system for case managers in Taiwan. Therefore, this study aimed to develop an IT-based support app to meet the needs of dementia care case managers in Taiwan. We believe the availability of an app that streamlines the organization of case managers’ responsibilities could improve the quality of care provided to persons living with dementia and their family caregivers in Taiwan.

## Methods

### Study Design

**Figure 1:**
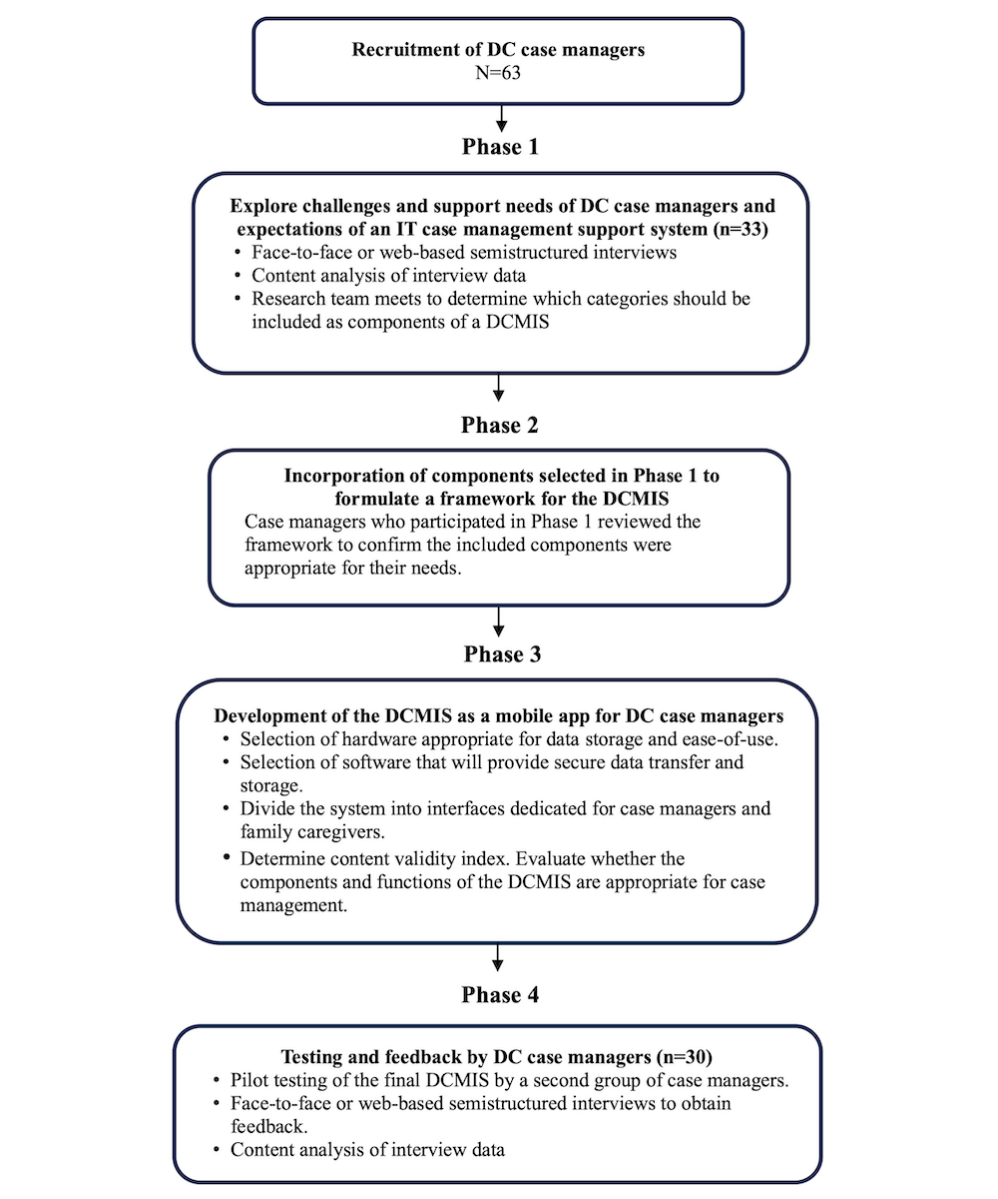
Overview of the study design. DC: dementia care; DCMIS: Dementia Case Management Information System.

This study was conducted between November 2020 and May 2023. The design of the study was implemented in four phases: phase 1 explored dementia care case managers’ perceived needs and their perspectives about IT support systems for dementia case management; phase 2 formulated the framework of a dementia case management information system (DCMIS) based on the findings of phase 1; phase 3 designed and developed the DCMIS for use as a mobile app and evaluated the validity of the system; and phase 4 pilot-tested the DCMIS app with dementia care case managers (users) and collected user feedback. Figure 1 provides an overview of the study design. The study is registered at ClinicalTrials.gov (NCT05131789).

### Participants

Case managers overseeing the coordination of care for persons with dementia at community-based facilities were recruited through purposive sampling from 28 facilities from shared dementia care centers and dementia care community service sites in Taiwan. Case managers were included if they met the following criteria: age 20 years or older, proficient in Mandarin or Taiwanese, and currently employed in one of the above dementia care facilities. There were no exclusion criteria. A total of 63 case managers were recruited to participate.

### Development of the DCMIS

#### Phase 1

During phase 1, semistructured interviews were either face-to-face or internet-based, using Google Meet, based on the preference of each participant. All interviews were conducted with one of two members of the research team (CYK and YLS). Questions followed a semistructured interview guide developed through discussions with members of the research team (Textbox 1). Interviews were recorded and transcribed verbatim. Analysis of interview data was conducted by three members of the research team (CYK, YLS, and HLH) using thematic analysis. Members of the research team met to discuss the findings and determine what identified components were described with the highest frequency by case managers. Any disagreements about what should be included or excluded were discussed and revised until a consensus was reached. Case managers who participated in phase 1 were invited to review the included items to determine whether the components of the DCMIS were relevant to the needs they expressed during the interviews.

**Semistructured interview guide for phase 1: perceived challenges and IT needs of dementia care case managers.**

**Duration, responsibilities, and experiences of being a case manager**

- How long have you been working as a dementia case manager?
- What are the main responsibilities in your role as a case manager?
- How do you feel about the process of performing your duties?

**Factors influencing job performance**

- What factors influence your performance as a case manager?
- Are there any resources available that could improve your performance as a case manager?

**Barriers to fulfilling the responsibilities of a case manager**

- Are there any aspects of being a case manager that you find challenging?
- (If yes): What type of assistance would help you navigate the challenge or challenges?

**Availability of IT systems**

- Do you use any IT system to support your work as a case manager?
- How does this help you with your work?
- Do you feel comfortable or uncomfortable using the system?

**IT systems that could improve case management for persons with dementia**

- Looking ahead, would you consider using an IT system to assist you in your role?
- Why would you be willing or not willing to use an IT system for support?
- Do you have any suggestions for such a system or the specific type of assistance you would hope would be provided?

**Regarding this interview, do you have any other thoughts or ideas you would like to share or discuss?**

#### Phase 2

Phase 2 involved formulating the framework of the DCMIS based on the components identified during phase 1. Dementia care case managers who participated in phase 1 were invited to review the components of the app to confirm whether they were appropriate and functional for case management of persons with dementia.

#### Phase 3

Phase 3 focused on the development of the DCMIS as a mobile app, which involved an interdisciplinary team comprised of an associate professor from the Department of Computer Science and Information Engineering, two IT engineers, and two experts in dementia care.

The DCMIS operating system was designed to use Microsoft Windows Server 2019 and was based on the LINE (LY Corporation) instant messaging platform. LINE is the most commonly used instant messaging app among Taiwanese, accounting for 77.56% [13]. We used the LINE messaging application programming interface to develop a LINE bot, which requires a webhook URL to receive webhook payloads from the LINE platform. For this purpose, we opted for a secure unified Ingress platform, ngrok [14], which is a proxy server enabling the creation of an HTTP/HTTPS server with a simple command. The ngrok platform was used to establish a secure tunnel with localhost on our local machine, allowing locally running services to be securely accessed over the internet through this tunnel. All responses from the bot server to users are programmed using Python codes stored in an Amazon Elastic Compute Cloud (EC2) virtual machine. The response content can be either text or a hyperlink. It was important that information entered by the users be secure and have cross-platform connectivity; therefore, Hypertext Preprocessor (PHP) scripting language was used, which allows the hyperlink to direct users to a web page where they can securely complete forms such as detailed patient information. The open-source database management system, MySQL, was configured to use the EC2 server for storing user information, patient data, questionnaire items, scoring correspondence tables, and any other needed data. To enhance network security on the EC2 virtual machine, all unnecessary ports were closed and connections for program updates were made using the default remote desktop protocol, which uses a 2048-bit SSH-2 RSA key pair for security. Database security was further enhanced by removing default accounts and using nonroot accounts, applying secure hash algorithm-1 encryption for sensitive data, and scheduling automatic backups.

During this development phase, biweekly meetings were conducted to assess progress, address challenges, and resolve system errors. We also sought consultations with software experts when necessary and feedback from the phase 1 case managers to ensure the DCMIS included all user needs.

Following the development of the DCMIS, four experts in dementia case management were invited to evaluate whether the content of the components and functions of the system were appropriate for case management. Each component was evaluated on a 4-point Likert scale: 1=very inappropriate; 2=inappropriate; 3=appropriate; and 4=highly appropriate. The content validity index (CVI) was calculated as the sum of the scores for each item divided by the number of experts. None of the items had a CVI ≤2, thus none were excluded. Although experts proposed renaming the case management function labeled, “Add/Select Case” to “Case List,” discussions with the research team believed these changes would not impact usability, and no change was made. The CVI for all components was > 0.9, indicating all were acceptable [15,16].

#### Phase 4

A second group of dementia care case managers participated in using the DCMIS. They then provided feedback on their experiences through face-to-face interviews as described for phase 1, but interviews were guided by a second semistructured interview guide (Textbox 2).

**Semistructured interview guide for phase 4 for case managers’ feedback about using the dementia case management information system (DCMIS) app (n=30).**

**Experience of using the DCMIS for case management**

- What was your overall impression of the app?
- Could you please share your opinions or reactions about the functionality of the components including in the DCMIS?

**Impact of the DCMIS on case management**

- Do you think the DCMIS with have a positive impact on your work as a case manager?
- Can you tell me more about that?

**Changes or modifications needed for the DCMIS**

- Do you think there are any additional functions or modifications that should be made to the DCMIS to improve case management?
- Why do you think these are needed?

**Other variables about the DCMIS**

- Are there any factors that might influence whether you use the DCMIS?
- Could you explain why?

**Do you have any other thoughts, ideas, or recommendations about the DCMIS app you would like to share?**

### Data Collection and Analysis

The collection of interview data and thematic analysis of transcribed interviews were conducted concurrently. Interview data were analyzed with thematic analysis using the method described by Braun and Clark [17]. During phase 1, text was identified to understand the key categories that described case managers’ experiences and perceived needs. During phase 4, text was identified that described the case managers’ experiences of using the DCMIS. For both phases, text was analyzed line-by-line to identify the experiences of the dementia care case managers and coding of the identified text was used to generate the key categories to provide an understanding of the experiences of the case managers [17,18]. The rigor of the interview data was maintained by the credibility of the data, which occurred by allowing other members of the research team to provide feedback about the validity of the analyzed data, and by dependability, which occurred by maintaining an audit trail of the process of data collection and analysis. Confirmability of the findings was provided by peer debriefing sessions; transferability was ensured by comparing the quantitative data with existing research on dementia case management [18].

### Ethical Considerations

This study was approved by our institutional review board (phase 1: IRB 202000902B0; phases 2-4: IRB 202100914B0). All case managers were informed of the study’s purpose, procedures, and potential risks before participating. Informed consent was obtained in writing from all participants. The data were anonymized, no personal identifiers were used in the analysis or reporting of the data, and participant confidentiality was strictly maintained. As a token of appreciation, all participants who completed the interview received a US $6 gift voucher.

## Results

### Participants

A total of 63 case managers were recruited to participate. Phase 1 included 33 case managers with a mean age of 36.7 (SD 10.9; range 23-63) years, most were female (n=29, 88%), and most (n=32, 97%) had a bachelor’s degree or higher. The average duration of employment as a dementia care case manager was 28 (SD 19.2; range 6-108) months. Phase 4 included 30 case managers with a mean age of 38.4 (SD 11.5; range 24-62) years, most were female (n=26, 87%), and most had a bachelor’s degree. The average duration of employment as a dementia care case manager was 43 (SD 35.6; range 4-156) months.

### Phase 1

#### Overview

Analysis of the interview data indicated that the challenges faced by dementia care case managers were high caseloads and a lack of support services. These were the result of increases in the number of persons with dementia, the time required to enter large amounts of patient data and administrative paperwork, and a lack of support services for themselves, as well as for persons with dementia and their family caregivers. The results of the analysis of the interview data are summarized in Textbox 3. The following information was provided when participants from phase 1 (P1) were asked about what challenges acted as barriers to fulfilling their role as a case manager.

**Challenges and IT needs for case managers (n=33).**

**Challenges**

- High caseload numbers and concomitant increases in paperwork
  Difficult to keep track of cases
  Missed follow-ups
- Support services disorganized and complicated to navigate
- Absence of an established health education database
  Additional time spent searching for material
  There is no source of material for caregivers
  No digital database requires carrying large amounts of hard copies
- Little internet-based sources for health education and case management
- Lack of remote data access about patient information

**IT needs**

- An integrated system with a user-friendly input mechanism
- A comprehensive database of available resources
  Type and description of dementia care services
  Type and description of support services
- A detailed health education model
- A communication system to improve patient handover
- A reminder function to identify cases needing assessments or follow-ups
- An itemized list of available dementia education materials for family caregivers

#### Challenges

One barrier mentioned by nearly all case managers was the significant increase in the size of their caseload. They also mentioned that manpower has not increased accordingly, and therefore, they often miss follow-ups, and the amount of paperwork has increased. Some case managers expressed feelings of powerlessness. One case manager contemplated resigning, saying:

Due to the large number of cases, it is impossible to track every case and understand their current status. I feel guilty about this. I have been angry with myself; at one point I considered resigning.

P1.CM01

A second barrier mentioned was the absence of a system that lists and describes available support services that address the care needs of persons with dementia. Several case managers mentioned this challenge. The following comment was typical:

To be honest, I’m not very familiar with community resources because there are so many types of services. There are dementia community service sites, long-term care stations, daycare services, small-scale multi-functional services, and respite care services. These things are just too diverse.

P1.CM16

Other comments included the following:

I’m not familiar with tasks like identification and certification of a disability or hiring a foreign caretaker. I need to study the application procedures and the required documents, which means considerable time spent searching for information.

P1.CM04

Case managers also found the lack of a health education database added to their frustration and inability to help patients navigate the system. Case managers described the need to invest additional time searching for health education information for themselves, as well as for patients and family caregivers. Another case manager mentioned family caregivers also found it challenging to obtain relevant information on their own, saying:

Some family caregivers don’t know where to find these resources and have already begun caring for a family member with dementia (without any information).

P1.CM26

The absence of internet-based sources of information about support services and health education material meant that case managers needed to carry large volumes of hard copies. One case manager said:

My colleague prints out all the health education materials and case information and puts them in a folder for easy access, but I feel it’s inconvenient to carry the folder around for work.

P1.CM21

#### Information Technology Needs

All participants agreed that an IT system would be useful for addressing some of the challenges that were barriers to fulfilling their role as case managers. Making patient data available internet-based would reduce the time required for case management. Although all case managers used the IT systems provided by the government, they expressed frustration that these were not adequate for supporting most of their case management tasks. Some case managers used available software spreadsheets to meet their needs. One participant provided the following description:

I believe effective case management requires multiple reminders. These are used as prompts for tracking specific cases on a given day, identify individuals requiring reassessment, and specifying the focus of the current follow-up. I have 20 individuals needing follow-up today. Without the use of an Excel spreadsheet, I would not know which patients will be present.

P1.CM02

When asked what should be included in an IT system designed specifically for dementia care case managers, first and foremost was the need for a user-friendly input mechanism. Regarding the components that should be considered, the following were mentioned most frequently: a comprehensive database of resources listing and describing available types of dementia care and support services, a detailed health education module, and a shared platform that would allow communication between team members, which could improve patient handover. Additionally, many case managers emphasized the necessity of reminder functions for efficient case follow-up. One participant explained:

At times, due to the demanding nature of our responsibilities, we may overlook pending tasks. With a reminder function, we would be able to identify cases yet to be assessed.

P1.CM17

Case managers also believed a health education component should be available, which could be provided to caregivers. Several suggested constructing a needs-specific list of available materials within the areas of dementia care. Case managers could then check a box to indicate materials of interest, which would make it easier to access educational material appropriate for caregivers of their cases. A case manager provided the following example:

If a case manager has a client with dementia who is exhibiting behavioral problems, they could check a box next to ‘behavioral problems’ to generate a list of available educational material that could be provided to the caregiver.

P1.CM20

### Phase 2

**Figure 2:**
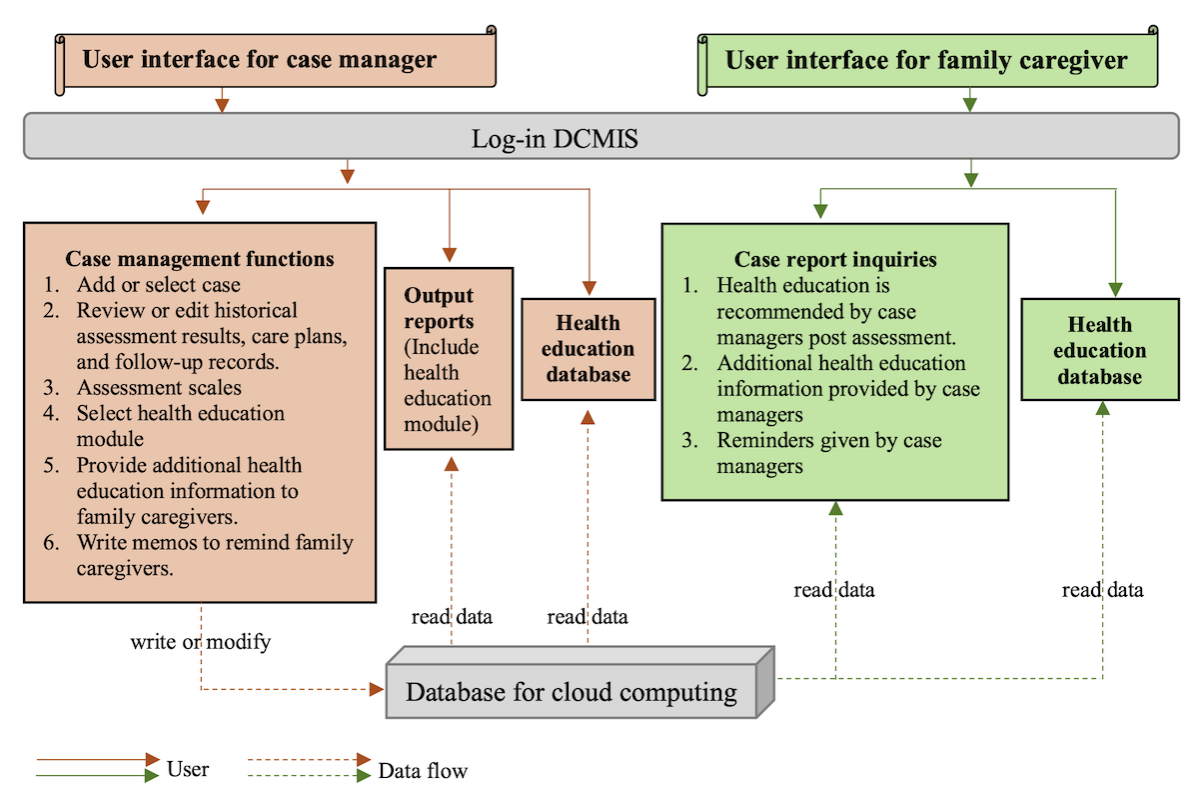
The framework of the DCMIS. DCMIS: Dementia Case Management Information System.

Based on the challenges and needs expressed by dementia care case managers, a framework for the DCMIS was developed. Because interview data showed case managers believed that there should be a component of the DCMIS that could be used by caregivers, the framework included an interface for case managers, as well as one for caregivers. A diagram of the framework of the DCMIS is shown in Figure 2, which includes a schematic of the case manager interface and theoretical interactions with a family caregiver interface.

The case manager interface was comprised of three blocks: case management functions, such as assessments, care plans, and reminders; output reports; and a health education database. The assessment block incorporates comprehensive assessment scales. When conducting case management, case managers can select relevant scales based on the needs of physical and mental assessments for the individuals or caregivers, for example, activities of daily living, brief version, and Chinese version of the Zarit Burden Interview (sCZBI-12). The care management follow-up block incorporates a case management database encompassing patient and family caregiver information, historical assessment results, care plans, and follow-up records. Case managers can review each case, provide care plans, and provide timely reminders for follow-ups. Historical case management data can be exported into reports for review or modification and synchronized with the caregiver interface, which has not yet undergone any clinical trials. The health education database integrates the research findings on dementia [19-24] and existing service resources, which incorporate an introduction to the different stages of dementia, treatments and care, family caregiver support, existing care resources, other chronic disease care, and common health issues. Dementia care case managers can directly select and query the required information from a database. Alternatively, after conducting case assessments, the system automatically and synchronously provides corresponding health education suggestions.

Each block of the case manager interface was designed to facilitate communication with their cases through a family caregiver interface. Although not yet tested with caregivers, the theoretical schematic is included in Figure 2, illustrating how family caregivers will be able to receive health education information, reminders, and follow-up records from the dementia care case managers. Additionally, caregivers will be able to autonomously search for relevant health education content using keywords. Given the prevalence of foreign caregivers hired to provide care for persons with dementia, specific health education materials will include versions in the languages of these caregivers.

### Phase 3

**Figure 3:**
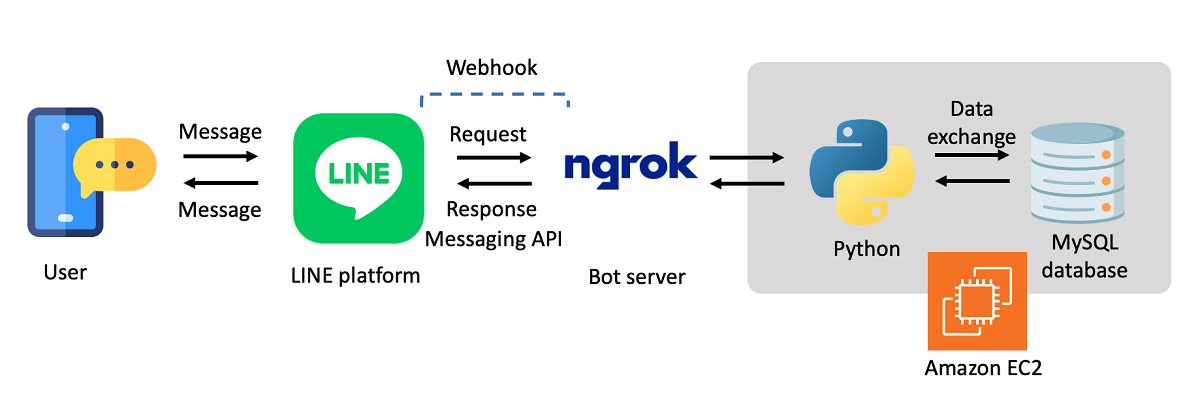
The final architecture of the DCMIS. API: application programming interface; DCMIS: Dementia Case Management Information System.

The final architecture of the DCMIS is shown in Figure 3. The use of the LINE mobile app, which is widely used throughout Asia [25], offers portability and freedom from environmental constraints. Case managers download the app to their smartphones, enabling them to perform assessments, health education, and case management. Further clarification and guidance about the use of the app were provided through instructional videos and user manuals.

### Phase 4

#### Overview

Analysis of interview data with case managers following pilot testing of the DCMIS app indicated that the overall experience was positive because the operation was user-friendly with only a few barriers. Three categories describe case managers’ user experiences using the DCMIS app: positive impact, facilitators of use, and barriers to use. Although the focus of pilot testing the DCMIS app was to get feedback about the interface for case managers, when asked if they had anything additional that they would like to share, case managers offered opinions about the caregiver interface, which involved two categories: anticipated benefits and anticipated difficulties. Components of these five categories are summarized in Textbox 4. Some of the specific comments from participants during the phase 4 (P4) interviews are provided below.

**Case managers experiences of pilot testing the dementia case management information system app and their opinions (n=30).**

**Case managers’ experiences**

- Positive impact
  User-friendly operation
  Enhanced accessibility of patient data
  Ease of tracking and recording data in real time
  Simplified interface and logical procedures
  Reduction in searching for paper-based educational materials
- Facilitators of use
  Components are time-saving solutions for high caseloads
    - Digital assessment tools
    - Streamlined case management
    - Comprehensive health education database
  Improved delivery of patient care and availability of information for caregivers
    - Automated reminders enhanced follow-ups
    - Improvements in handovers
    - Comprehensive care information about the trajectory of dementia
- Barriers to use
  Lack of integration of the DCMIS with the government operating system, which may increase redundancy
  Availability of the DCMIS as only a mobile app
    - Some case managers preferred using a desktop for data entry; no desktop option
    - Caregivers might view using a mobile app during consultations as impolite or unprofessional

**Case managers’ opinions about the caregiver interface**

- Anticipated benefits
  Information about the experience of living with dementia
  A better understanding of caregiving needs for persons living with dementia
  The ability to access educational material at a time convenient for the caregiver
- Anticipated difficulties
  Older caregivers might find it challenging to use a mobile app independently
  Some caregivers prefer being provided with information rather than searching themselves
  Oral- or paper-based health educational material might be desired by older caregivers

#### Positive Impact

Incorporating the DCMIS into a mobile app enhanced accessibility to patient data by enabling case managers to seamlessly access and track case information and record data in real-time without temporal or spatial constraints. In addition to the ease of use, the simplified interface and logical procedures were the key factors influencing case managers’ readiness to embrace the system. All case managers viewed that eliminating the need to search for supplementary paper-based health education materials reduced their workload considerably. One case manager appreciated being able to perform case management more easily:

I feel the app is very convenient. The ability to use my mobile phone with LINE allows me to perform tasks like record keeping and track the status of my cases anywhere, which reduces my workload tremendously. I don’t need to bring paper copies with me anymore! Most of my important work as a case manager is away from the office. The app allows to conduct record keeping away from my desk. I think it is very helpful.

P4.CM11

Another case manager described why the app was easy to use:

The interface is clear, so it’s easy to use. Although it took a little time to for me to adjust, when I became familiar with the interface, I understood the logic of it. Once this happened, it was very convenient to use. Including the ADL function is really a time-saver because it calculates the ADL score, which is not always simple.

P4.CM03

#### Facilitators of Use

Case managers reported that the DCMIS would facilitate significant savings in time. They felt the digital assessment tool had a positive impact on work efficiency, which would streamline case management when caseloads were high. The comprehensive health education database was also seen as a time-saving component because it would facilitate searching through stacks of documents and brochures. The generation of corresponding educational modules postassessment content was also considered a time-saver with real-time information delivery to family caregivers ensuring information was received without a delay and the generation of automated reminders from case managers to caregivers would enhance effective case follow-up. One case manager commented on the expectation that the app would improve handovers:

I find the presentation format of the output reports very clear. It systematically organizes and presents data and provides a transparent record of actions during the service process.

P4.CM21

Another case manager thought that the DCMIS app addressed the complexity of management:

Dementia care issues vary at each stage and every condition is unique. This system provides comprehensive care information covering the entire disease trajectory. This includes information that can be shared with family caregivers, which is highly beneficial to our work.

P4.CM04

#### Barriers to Use

One barrier to use that was expressed by some of the case managers was the lack of integration between the government’s operating system and the DCMIS. This was a concern because monthly entries into the government system are required for the registration of cases and reimbursements for services delivered. Case managers saw a potential for duplication of efforts if the systems were not integrated. One case manager said:

If the DCMIS is not integrated with the Ministry of Health and Welfare’s system, my data will need to be entered a second time, which will add redundancy to the system.

P4.CM28

The second barrier was that the DCMIS was only available in a mobile version. Some case managers mentioned they preferred a larger desktop system over a small mobile app. Not only was size a consideration, but they also believed that operating a mobile app while simultaneously engaging in clinical responsibilities or having a conversation with a caregiver might have a negative impact on their image as a professional. This was voiced by one case manager, who said:

If we are constantly looking at our mobile phones during a consultation, would the family caregivers perceive us as impolite?

P4.CM13

#### Anticipated Benefits for Family Caregiver Users

Case managers mentioned that the DCMIS could enable family caregivers to better understand the experience of dementia for their family members. One case manager said:

It could provide family caregivers with information about the current condition of their family member with dementia, which could provide caregivers with a better understanding of the caregiving tasks required.

P4.CM26

Another case manager said:

Family caregivers can simultaneously access caregiving recommendations or health education information on their mobile phones, which be convenient to reading during their free time.

P4.CM22

#### Anticipated Difficulties for Family Caregiver Users

Some case managers expressed concern that family caregivers might not be able to access and read health education information using an app without the help of someone else. One case manager believed older caregivers might find it challenging to use new technology, saying:

I think sometimes family caregivers prefer having information provided to them directly rather than having to do a search themselves, unless they are particularly diligent.

P4.CM03

Another case manager had the same concern, although they thought caregivers who were younger would be more willing to use the app:

The DCMIS interface may be suitable for younger or middle-aged caregivers familiar with mobile phone use. However, oral- or paper-based health education may be more appropriate for older adult caregivers.

P4.CM14

## Discussion

### Principal Findings

Development of the DCMIS app for dementia care case managers used a multistage process. The framework was designed to meet the needs of case managers, which was guided by the literature on eHealth, as well as qualitative interview data from 33 experienced case managers. The components of the app were examined for content validity and the usability of the app was pilot-tested with hands-on use by 30 case managers. Qualitative interview data provided feedback about functionality and how well the available components aligned with the assistance needed by case managers to benefit their job responsibilities.

The effectiveness and usability of the newly developed eHealth system increases when feedback is obtained from stakeholders who will be the primary users [26-28]. We explored the opinions of case managers regarding not only the type of help the DCMIS should provide but also what they expected in an eHealth app. Firsthand experience of case managers and qualitative interviews following sustained use of the app confirmed the app was feasible for use in a clinical setting.

The incorporation of the LINE messenger app into the system enhanced usability. Most people in Taiwan are already familiar with how to connect to the internet via their mobile phones through LINE messenger, which allows rapid exchange of information and services including text, images and video sharing, and searches [29-31]. This familiarity with LINE further enhanced usability because it was easy for case managers to record, query, and share information. The health education database incorporated into the DCMIS addressed case managers’ concern about the problem of time wasted when they needed to manually search through paper-based reports for information on dementia. Finally, the feature of separate interfaces for case managers and family caregivers was designed to allow synchronization of results of assessments and related health education measures allowing caregivers immediate access to patient information before meeting with the case manager. System feedback and individualization are functionalities strongly supported by participants [28,32].

Many of the available platforms have similar functions; however, their applications differ from the DCMIS app for dementia care case managers. Several apps provide support for family caregivers of persons with dementia [9-11,33], video consults and decision support tools to help dementia care nurses and case managers care for persons with dementia in residential care facilities [12], and internet-based training and support for informal dementia carers [28]. An app with similar functions to the DCMIS app is available in Taiwan but its purpose is to provide nurses with tracking information related to location and fall detection for persons with dementia residing in nursing homes [34]. By contrast, the DCMIS app was developed to streamline the work performed by dementia care case managers with a design focused on making it easier to implement dementia case management practices and improving the quality of care for persons with dementia.

The ability of the DCMIS app to help with tracking assessments, care plans, and follow-up reminders, and the inclusion of a health education database will allow integration of the platform with other health systems in Taiwan. Future expansion of the DCMIS to other health care facilities should increase the efficiency of scheduling patient assessments, follow-ups, and receiving test results that require an exchange of information between dementia care case managers and dementia care medical centers, community-based clinics, or home health care providers. Dementia and health care–related assessment tools for behavioral problems, sleep quality, frailty, nutrition, and activities of daily living can be reviewed and associated health education suggestions can be generated. The design of the DCMIS app makes it easier to manage large case numbers by including reminders about when patients need critical assessments or follow-ups so that appointments are not missed. This function will be essential during periods of unexpected reductions in manpower, as occurred during the COVID-19 pandemic. A loss of manpower results in a higher-than-normal caseload, and this reminder function minimizes errors that occur when appointments and follow-ups are paper-based.

The DCMIS app enables case managers to complete multiple tasks on one platform, thus enhancing work efficiency. It is hoped that the system will eventually include an interface with a caregiver app, which will further increase continuity of care. Case managers’ concerns that older caregivers of persons with dementia might find it difficult to interact with case managers via an app suggests it will be important to ensure user-friendliness for all ages and abilities when the caregiver app is launched. It is believed this issue will be ameliorated by alterations in the design through interdisciplinary collaborations [35]. Future applications could also include training of current case managers or nursing students.

### Limitations

This study had several limitations. First, as mentioned, integrating the DCMIS with the government’s operating system to avoid redundancy might not be achievable due to the barriers of integrating the system with Taiwan’s IT system, which is based on financial reimbursements. This is in sharp contrast to the DCMIS, which was developed as a tool to improve case management and improve continuity of care. Second, the current DCMIS is only available as an app for mobile phones; it is unclear when it will be available for desktop computers. The convenience of simultaneous assessment and recording was appreciated by some case managers but others were concerned the use of the mobile app might be perceived as disrespectful. Future designs should consider user-friendliness and diversity to align with individual preferences and use habits. Finally, family caregivers were not included in any of the phases for input into their needs or pilot testing of the app and providing feedback, which was due to lack of time and manpower. Future research should include family caregivers to promote better alignment of the DCMIS with their needs.

### Conclusions

The growing aging population and increase in the number of persons with dementia present a global challenge to the adequacy of care resources. The availability of a case management app could improve the management of health care for persons with dementia by improving efficiency when managers are faced with high caseloads. Participants who reacted positively to the DCMIS app did so because it was easy to use, and they perceived their caseloads were easier to manage. Our findings suggest that the incorporation of eHealth could enhance efficiency for case managers and improve the quality of care provided to persons with dementia. Our findings can serve as a reference for future research on the development and application of eHealth systems for other case managers. However, a large-scale clinical trial with a larger number of dementia care case managers and multiple health care systems will need to be conducted to quantitatively assess the effectiveness of the DCMIS app on case management outcomes including improvements in patient care and support for family caregivers.

## Abbreviations

CVI: content validity index
DCMIS: dementia case management information system
